# Intensity Thresholds and Maximal Lactate Steady State in Small Muscle Group Exercise

**DOI:** 10.3390/sports8060077

**Published:** 2020-05-28

**Authors:** Florian Spendier, Alexander Müller, Markus Korinek, Peter Hofmann

**Affiliations:** 1 Exercise Physiology, Training & Training Therapy Research Group, Institute of Sports Science, University of Graz, 8010 Graz, Austria; florian.spendier@edu.fh-joanneum.at (F.S.); alexander.mueller@uni-graz.at (A.M.); markuskorinek@gmail.com (M.K.); 2 Division of Endocrinology and Diabetology, Department of Internal Medicine, Medical University of Graz, 8036 Graz, Austria

**Keywords:** maximal lactate steady-state, biceps curl exercise, incremental exercise, constant-load exercise, lactate shuttle theory

## Abstract

The aim of our study is to determine the first (LTP_1_) and the second (LTP_2_) lactate turn points during an incremental bicep curl test and to verify these turn points by ventilatory turn points (VT_1_ and VT_2_) and constant-load exercise tests. Twelve subjects performed a one-arm incremental bicep curl exercise (IET) after a one repetition maximum (1RM) test to calculate the step rate for the incremental exercise (1RM/45). Workload was increased every min at a rate of 30 reps/min until maximum. To verify LTPs, VT_1_ and VT_2_ were determined from spirometric data, and 30 min constant-load tests (CL) were performed at 5% P_max_ below and above turn points. Peak load in IET was 5.3 ± 0.9 kg (La_max_: 2.20 ± 0.40 mmol·L^−1^; HR_max_: 135 ± 15 b·min^−1^; VO_2max_: 1.15 ± 0.30 L·min^−1^). LTP_1_ was detected at 1.9 ± 0.6 kg (La: 0.86 ± 0.36 mmol·L^−1^; HR 90 ± 13 b·min^−1^; VO_2_: 0.50 ± 0.05 L·min^−1^) and LTP_2_ at 3.8 ± 0.7 kg (La: 1.38 ± 0.37 mmol·L^−1^; 106 ± 10 b·min^−1^; VO_2_: 0.62 ± 0.11 L·min^−1^). Constant-load tests showed a lactate steady-state in all tests except above LTP_2_, with early termination after 16.5 ± 9.1 min. LTP_1_ and LTP_2_ could be determined in IET, which were not significantly different from VT_1_/VT_2_. Constant-load exercise validated the three-phase concept, and a steady-state was found at resting values below VT_1_ and in all other tests except above LTP_2_. It is suggested that the three-phase model is also applicable to small muscle group exercise.

## 1. Introduction

The lactate shuttle theory [1,2,3] and the triphasic model of energy supply [4] have been extensively prescribed and were proven to be valid for cycle ergometer exercise [5], but has not been implemented yet in sports accordingly [6]. In short, the theory prescribes three phases, usually prescribed as aerobic, aerobic–anaerobic transition, and anaerobic phases, separated by to thresholds, although the terms aerobic and anaerobic are misleading [7]. Several authors have, therefore, proposed to use the terms first and second threshold or turn point [5,8] to avoid misunderstanding. The main principle is the balance between lactate production and oxidation/elimination, the lactate shuttle theory [1,2,3]. For a better understanding of the interplay between local muscle and whole-body metabolism in small muscle incremental exercise, we, therefore, employed the lactate turn point concept [4,8,9,10].

This concept [4,8,9,10] defines a metabolically balanced phase on local muscle level (phase I) below the first threshold, a systemically balanced metabolic phase (phase II) between thresholds 1 and 2 and a phase with no metabolically balanced situation (phase III) above threshold 2 [11]. Regarding Brooks [3], lactate is also produced under systemic aerobic conditions but is metabolized locally in the muscle cells and thereby cannot be measured systemically. It follows that such a three-phase energy supply cannot be strictly limited to aerobic and anaerobic areas, but a mixing ratio always occurs [7].

The question arises if the same patterns and principles, as shown for large muscle group cycling exercise [4,8,12], may also be verified for very small muscle group incremental exercise. Therefore, we investigated metabolic and cardiorespiratory parameters during unilateral biceps curl exercise.

Some recent articles investigated the phenomenon for incremental and continuous resistance exercise and their physiological patterns [13,14,15,16,17,18,19,20,21,22,23,24,25,26]. These studies focused on resistance exercise of lower limbs, like leg press or half-squat exercise. Initially, a one repetition maximum (1RM) test was conducted, which served as the basis for the intensity of the incremental resistance exercise. All incremental resistance exercise tests were performed discontinuously, with resting sets in between effort intervals. Several authors have evaluated the responses that occur after a resistance training session at lactate threshold (LT) intensity [14,17,18,19,20,25]. LT occurred at exercise intensities ranging from 27% to 36% of 1RM [16], a value that can be attributed to physiological and hemodynamic mechanisms [14]. Heart rate, lactate progression, and ventilatory parameters (VO_2_, VCO_2_, VE) stabilized during constant-load exercises at LT intensity, equivalent to lactate turn point 2 (LTP_2_) intensity, although there was no consistent definition of threshold intensities applied. The terms LT [13,14,15,17,18,19,20,21,23,26] and anaerobic threshold (AT) [24,25] were used synonymously. Blood lactate concentrations and heart rate values during constant-load exercise were higher in half-squat than reported in leg press exercise [16].

Although the principal pattern has been prescribed, including threshold determination, validation of the concept of applying steady-state exercise has not been shown yet. Our study is the first to display lactate turn point 1 (LTP_1_) and ventilatory threshold 1 (VT_1_) determination during incremental small muscle exercise by means of systemic measures. For this reason, the aim of this study is to determine the first (LTP_1_) and the second (LTP_2_) lactate turn points during an incremental biceps curl test and to verify these turn points by ventilatory turn points (VT_1_ and VT_2_) and constant-load exercise tests. We hypothesize no significant difference between lactate and ventilation derived thresholds in small muscle group exercise and that these thresholds can be verified by lactate steady-state tests.

This specific type of performance diagnostic could provide a new perspective on a more generalized model of training prescription, although additional studies, including different muscle groups, are still needed. The answer to this question is of considerable importance in the field of work physiology, as local muscle performance diagnostics functions as a tool to avoid overloading workers and prevent reduced performance levels associated with physical/mental damage [27].

## 2. Materials and Methods

The study included a total of 12 healthy sports students (10 men, 2 women; age: 24.8 ± 3.1 years; height: 181.4 ± 7.3 cm; body mass: 76.0 ± 8.1 kg, cycle ergometer VO_2max_: 51.05 ± 5.90 mL·kg·min^−1^; 1RM: 14.46 ± 3.77 kg) (Table 1). The subjects performed an incremental and four constant-load biceps curl tests. The number of subjects required to make a statistically relevant statement was determined using G * Power Data Analysis Examples program [28]. All subjects gave their informed consent for inclusion before they participated in the study. The study was conducted in accordance with the Declaration of Helsinki, and the protocol was approved by the Ethics Committee of the University of Graz (GZ 39/48/63 ex 2015/16). All data were collected in the laboratory under standardized conditions.

### 2.1. Used Devices and Measuring Methods

The small muscle group exercise measurements were carried out on an adjustable weight training bench. The adjustable part of the bench functioned as a supportive surface for the upper arm, in order to perform an isolated strain on the m. biceps. All tests were done with the dominant arm. All participants were instructed to keep their non-dominant arm in an inactive hanging position to avoid interference with the dominant arm. Subjects completed the tests seated, and a height-adjustable seat was individually accommodated (Levels 1–15). Free weights (dumbbells) were used for the tests, and the weights were symmetrically attached. Lactate was determined from the arterialized capillary blood of the ear. The first drop of blood was removed. The samples taken were evaluated immediately after each test with a Biosen S_line device (EKF Diagnostics GmbH, Barleben, Germany) to determine blood lactate and blood glucose concentrations. The determination of individual turn points from blood lactate values was carried out by means of linear regression breakpoint analysis using proSport performance diagnostics software [29].

The mobile device Cortex MetaMax 3B (Cortex Biophysik GmbH, Leipzig, Germany) was used to measure gas exchange variables. Before each use, the measurement device was calibrated according to the manufacturer’s instructions. Initially, the data were collected using the program Cortex Metamax 3B Version MBX 3B2.1, Metasoft 3.9, and then merged with proSport to determine VT_1_ and VT_2_ similar to LTP_1_ and LTP_2_.

### 2.2. Data Analysis and Turn Point Determination

All data were evaluated by means of Microsoft Excel (Microsoft, Redmond, WA, USA), in which the individual measured parameters of the different tests were assigned to the subjects’ identification number. Thus, the mean values and standard deviations (±SD) could be calculated for random times. During the incremental-step tests and the constant-load tests, heart rate was recorded continuously and stored in five-second intervals using a standard heart rate monitor (Polar Electro, S810i, Kempele, Finland). The heart rate data were transmitted and evaluated via an infrared interface to the performance diagnostics software proSport, as prescribed earlier [29]. The turn points obtained from the incremental biceps curl test were used to set the intensity of the subsequent constant-load tests. First (LTP_1_) and second lactate turn points (LTP_2_) were identified by a linear regression breakpoint analysis. Each LTP_1_ and LTP_2_ was determined as the intersection points between two regression lines within defined regions of interest, such as between first load step and 60% of maximal power during incremental biceps curl test (P_max_) and between first turn point and P_max_. Additionally, the first (VT_1_) and the second (VT_2_) ventilatory thresholds were determined by applying the same linear regression breakpoint analysis [29] using the proSport performance diagnostics software. 

### 2.3. Test Procedures

#### 2.3.1. One Repetition Maximum (1RM)

Before the incremental and the constant-load tests, a determination of the one repetition maximum (1RM) was carried out for all subjects. The range of motion of the bicep curl was set at maximum extension in the elbow joint (180°) and flexion of 90° so that permanent muscle tension was given. Prior to the maximum load, an extensive warm-up of the working muscle at 50% of the expected maximum with ten repetitions took place. Following the warm-up, another submaximal set of 70–80% of the expected maximum was performed to accustom the muscle to the one-repetition-maximum test. Based on the previous experience of the subject or at the discretion of the test leader, a corresponding weight was chosen. If it could be performed properly, the load was increased in order to determine the 1RM with a maximum of six attempts. There was a five-minute break between each attempt for optimal recovery. The 1RM was used to determine the expected maximum for the incremental test, as well as the load increments. During the 1RM determination, only the load (in kg) was determined, and no physiological measures were performed.

#### 2.3.2. Incremental Bicep Curl Test

The expected maximum of the gradually increasing load was suggested approximately between 30% and 40% of the 1RM, according to the literature [14,25]. 

**Figure 1:**
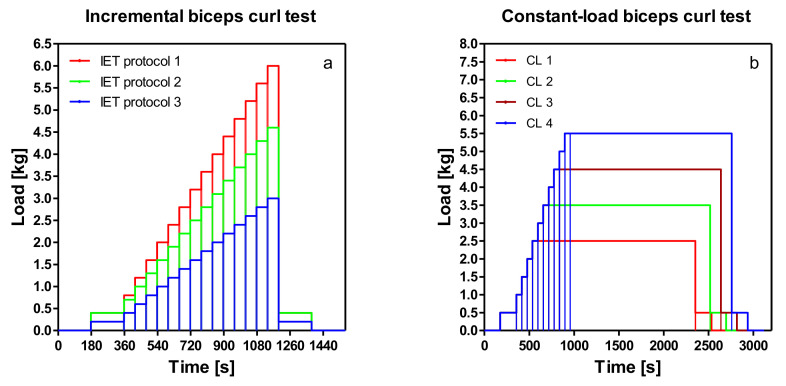
(**a**) Schematic illustration of incremental bicep curl test (IET) and (**b**) constant-load bicep curl tests. CL1—5% P_max_ < LTP_1_; CL2—5% P_max_ > LTP_1_; CL3—5% P_max_ < LTP_2_; CL4—5% P_max_ > LTP_2_.

As for the subsequent lactate turn point determination, about 15 samples were required; the individually achieved 1RM was divided by 3, and this calculated maximum for the incremental test was again divided by 15 to obtain the corresponding load increments. At the beginning of the test, the volunteers sat quietly without exercising for three minutes. Thus, the first measure reflects resting conditions for all variables. After the resting phase, a three-minute warm-up started with a weight that was chosen based on the result of the 1RM. Different increments were applied dependent on 1RM performance to guarantee the same overall test duration to reach maximal performance and a similar number of lactate measures to determine thresholds. Workload was increased every minute by 0.2, 0.3, or 0.4 kg until termination due to fatigue (Figure 1a). Adjustable dumbbells were prepared with exact load increments and were supported every minute to guarantee a non-paused exercise. The execution speed was two seconds (1 s for flexion, 1 s for extension of the forearm, 30 repetitions/min). Termination criteria were an incorrect execution of the movement, non-compliance with the execution speed, or inability (total fatigue) to continue the exercise. After termination of exercise, a cool-down phase was initiated where exercise was continued for another 3 min with the initial weight. The last lactate measurement took place after a further resting phase of 3 min. Lactate concentration was measured after each load step (1 min intervals) and immediately after termination of exercise. Heart rate and spirometric data were measured continuously. There were no resting intervals during the incremental step test.

#### 2.3.3. Constant-Load Tests

A total of four constant-load tests in randomized order were completed. The intensity specification of the tests was carried out using the lactate turn point model [4]. Constant-load tests were performed 5% of P_max_ below and above each turn point (LTP_1_, LTP_2_). These tests were performed in the same way as the incremental step test until the target load was reached and then kept constant for 30 min (Figure 1b). Heart rate (HR), blood lactate concentration (La), and gas exchange variables were measured. The tests were conducted without resting intervals.

#### 2.3.4. Statistical Analysis

Mean values ± standard deviation (± SD) were calculated for all individual parameters. After screening the data for Gaussian distribution (Kolmogorov–Smirnov test), a *t*-test for dependent samples was conducted. For non-normally distributed data, the Wilcoxon test was used. The relationship between the two variables was determined by linear regression analysis. The significance limit was assumed to be *p* ≤ 0.05. Statistical evaluation and the graphical representation were issued by the program GraphPad Prism 5 (Graph Pad Software, San Diego, CA, USA).

## 3. Results

### 3.1. Incremental Bicep Curl Test

**Figure 2:**
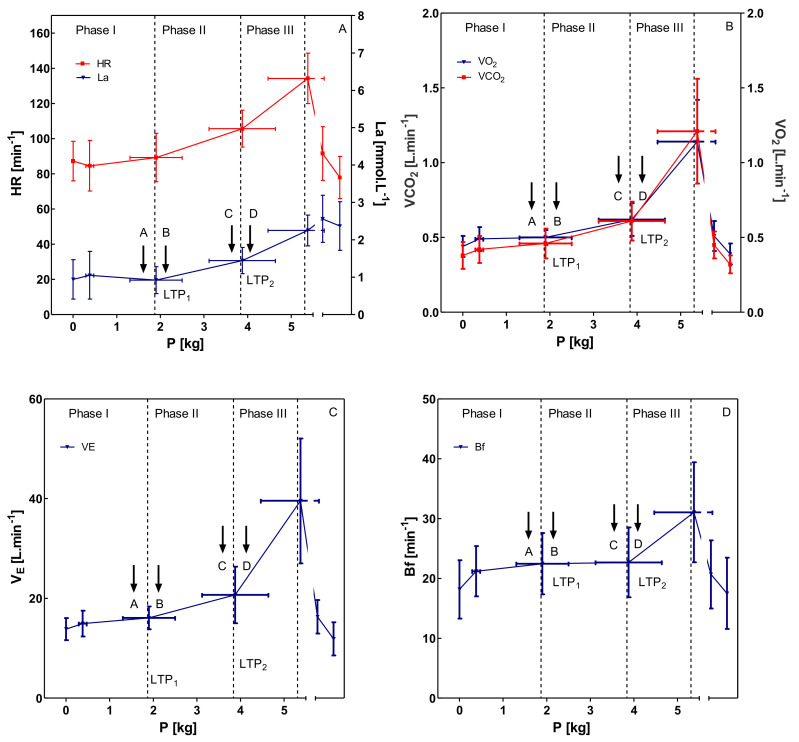
All data are presented as mean ± SD, calculated from all participants. Selected variables (HR—heart rate; La—blood lactate concentration; VO_2_—oxygen uptake; VCO_2_—carbon dioxide output; VE—ventilation; B_f_—breathing frequency) during an incremental bicep exercise test. Sub-maximal markers are the first and second lactate turn point. Exercise intensities for constant-load tests ((**A**)—5% of P_max_ < LTP_1_; (**B**)—5% of P_max_ > LTP_1_; (**C**)—5% of P_max_ < LTP_2_; (**D**)—5% of P_max_ > LTP_2_) and distinct phases (Phases I–II–III) are shown. Phase I: no increase in blood lactate concentration above baseline during constant-load exercise. Phase II: slight increase in blood lactate concentration. Phase III: strong increase of blood lactate concentration up to termination of exercise.

It was possible to detect a three-phase energy supply pattern and thus to determine submaximal parameters [4]. Figure 2 shows heart rate and blood lactate concentration (A), as well as oxygen uptake (VO_2_) and carbon dioxide output (VCO_2_) (B), ventilation (VE) (C) and breathing frequency (B_f_) (D) during the incremental biceps curl test. Heart rate showed an inverted deflection of the heart rate performance curve [14,30].

LTP_1_ was determined at a load of 1.87 ± 0.57 kg and LTP_2_ at a load of 3.84 ± 0.71 kg. Compared to the 1RM (14.68 ± 3.52 kg), LTP_1_ was 12.74% of 1RM, LTP_2_ 26.16% 1RM, and P_max_ 36.17% of 1RM. In terms of the maximum power delivered in the incremental bicep curl test (5.31 ± 0.86 kg), the percentages of LTP_1_ and LTP_2_ were calculated at 35.22% of P_max_ (LTP_1_) and 72.32% of P_max_ (LTP_2_).

Ventilation (VE) (C) and breathing frequency (B_f_) (D) showed an expected pattern which allowed to determine the ventilatory thresholds VT_1_ and VT_2_. From the first threshold (VT_1_) at 1.90 ± 0.56 kg (12.94% 1RM), the first increase in ventilation can be observed, and VT_2_ was determined at 25.20% of the 1RM and a load of 3.70 ± 0.57 kg. Maximal lactate concentration at the termination of the test was found at 2.25 ± 0.41 mmol·L^−1^. Both LTP_1_ and VT_1_ (r = 0.673; *p* < 0.001) as well as LTP_2_ and VT_2_ (r = 0.931; *p* < 0.001) were significantly related and a *t*-test revealed no significant differences between LTP_1_ and VT_1_ (*p* > 0.05) and LTP_2_ and VT_2_ (*p* > 0.05).

### 3.2. Constant-Load Bicep Curl Tests

**Figure 3:**
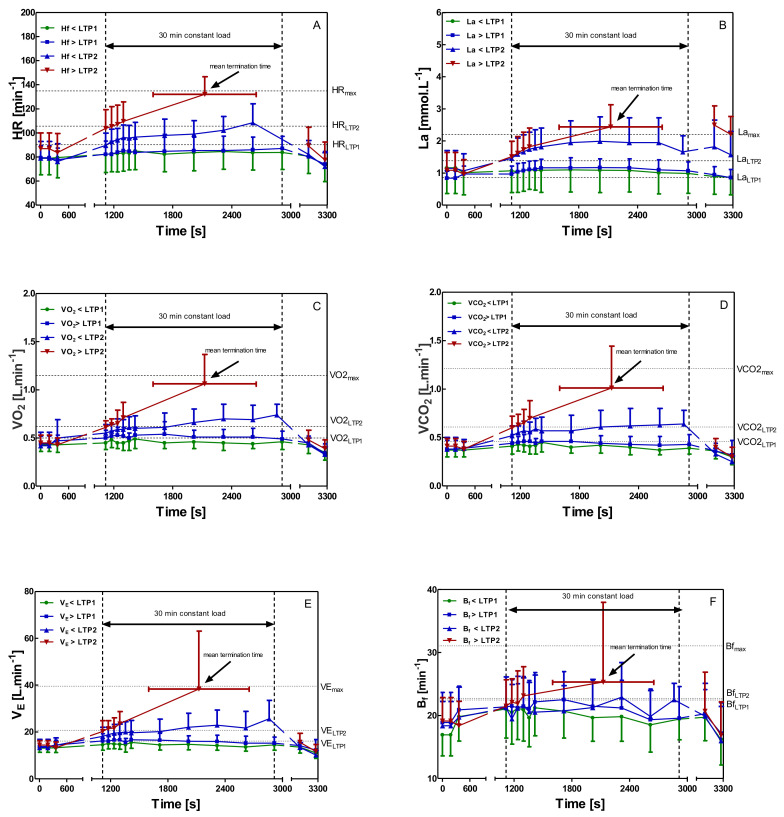
All data are presented as mean ± SD, calculated from all participants. Selected variables during constant-load exercise at 5% of P_max_ < LTP_1_, 5% of P_max_ > LTP_1_, 5% of P_max_ < LTP_2_, and 5% of P_max_ > LTP_2_ showing distinct phases (Phases I–II–III) of energy supply. Phase I: no relevant increase of blood lactate concentration above baseline during constant-load exercise, Phase 2: increased but steady-state blood lactate concentration during constant-load exercise, and Phase 3: continuous increase of blood lactate concentration leading to early termination of exercise. (HR—heart rate (**A**); La—blood lactate concentration (**B**); VO_2_—oxygen uptake (**C**); VCO_2_—carbon dioxide output (**D**); VE—ventilation (**E**); B_f_—breathing frequency (**F**))**.**

Heart rate during constant-load tests 5% of P_max_ below/above LTP_1_ differed only marginally, and both represent a steady state of the heart rate. Constant-load test 5% of P_max_ below LTP_2_ gave a heart rate increase of about 10 beats during the 30 min continuous load. The constant-load test 5% of P_max_ above LTP_2_ had to be terminated already at 16.83 ± 8.77 min of workout, reaching HR_max_ from the IET (Figure 3A).

Blood lactate concentration showed a lactate steady-state in all tests below LTP_2_ intensity, but a continuous increase in lactate and an early termination after 16.83 ± 8.77 min was found for a workload above LTP_2_. Small increases in systemic lactate concentrations from baseline were found such as delta lactate values of −0.17 ± 0.27 mmol·L^−1^ (<LTP_1_), 0.13 ± 0.41 mmol·L^−1^ (>LTP_1_), 0.79 ± 0.72 mmol·L^−1^ (<LTP_2_) and La increased by 1.36 ± 0.63 mmol·L^−1^ at a workload > LTP_2_ already within 16.83 ± 8.77 min (Figure 3B).

Figure 3C,D shows oxygen uptake and carbon dioxide output during the four constant load tests. Oxygen uptake and VCO_2_ remain at resting values during the whole constant-load exercise, and no respiratory compensation was identifiable for the exercise below LTP_1_. A workload above LTP_1_ initially increased VO_2_ and VCO_2_ but continued in a steady-state similar to the workload below LTP_2_, where VO_2_ and VCO_2_ slightly increased, but VO_2_ was higher than VCO_2_ during the entire test duration. Above LTP_2_, VCO_2_ concentration finally reached the same value as the VO_2_, and constant load exercise had to be stopped.

Depending on the level of intensity of the constant-load exercise, ventilation (Figure 3E) and breathing frequency (Figure 3F) showed an initial increase with a steady-state pattern until termination. The constant-load exercise above LTP_2_ had to be terminated early, and VE and B_f_ showed a rapid increase with maximum values of 38.36 ± 24.73 L·min^−1^ (VE) and 25.31 ± 12.63 min^−1^ (B_f_).

## 4. Discussion

The major findings of this study were that it was possible to verify a three-phase energy supply model in our small muscle group incremental biceps exercise tests, to determine submaximal markers for lactate (LTP_1_, LTP_2_) and ventilation (VT_1_, VT_2_) [4,8], and to validate these markers by means of lactate steady-state tests. The turn points for La and VE were significantly related and not significantly different. All workloads below LTP_2_ gave a clear steady-state for lactate, but La increased significantly, and workload had to be terminated early for exercise above LTP_2_ validating the applied threshold concept.

LTP_1_ and the LTP_2_ were determined at 13.14% ± 4.15% 1RM, and 26.83% ± 5.26% 1RM which is comparable to former work with leg press exercise at LTP_2_ intensity (LT, AT) at 27.8% ± 3.6% of 1RM [13], 27.1% ± 3.7% of 1RM [14], 31.0% ± 5.3% of 1RM [21], 28.0% ± 4.0% of 1RM [25], 29.0% ± 6.0% of 1RM [24], and 30.0% ± 6.2% of 1RM [23]. Several authors detected also thresholds (LT, AT) comparable to our LTP_2_ intensity in incremental half-squat exercise at 23.35% ± 4.32% of 1RM [17,18], 22.8% ± 3.54% of 1RM [17,18], 24.8% ± 4.8% of 1RM [19], and 24.8% ± 4.8% of 1 RM [20]. These small differences are suggested to be related to the involved muscle mass (biceps curl vs. leg press/half-squat), respectively, to continuous vs. discontinuous exercise or the use of different increments. However, the absolute systemic lactate levels (biceps curl) differ from similar work [17,18,19,20] due to the greater dilution of the lower absolute amount of lactate produced by the small biceps muscle mass compared to larger muscles such as shown for leg exercise [31]. Muscle biopsies or tracer techniques could give a more detailed analysis of the muscle to system interaction for the different muscle masses involved (e.g., biceps vs. leg press). We suggest, however, that independent from muscle mass, the same methodological approach, as shown here, should be applied to guarantee an accurate comparison of data. In recent work regarding leg press [13,14,21,23,24,25], higher intensities as a % of 1RM during the incremental tests were applied than for half-squat exercise [17,18,19,20]. Leg press reached up to maximal incremental intensities of 60% of 1RM and half-squat exercise up to 40% of 1RM [16]. However, no comparable results similar to our LTP_1_ intensity could be found.

LTP_2_ was found at 26.83 ± 5.26% 1RM, which is at the lower range prescribed in previous studies [13,14,21,23,24,25]. Beside limits in the oxidative capacity of the muscle, local hemodynamic constraints may limit the working muscles. Due to increasing intramuscular pressure from a load of approximately 25% of the 1RM, blood flow is suggested to be reduced significantly, limiting the oxygen supply. It was shown that there is no total occlusion in conduit arteries, but blood flow in the capillary bed was highly reduced at 20% of maximum voluntary contraction (MVC) [32]. Additionally, it was pointed out that intramuscular pressure rose with increases in contraction force [33] and was found to restrict blood flow during contractions above 25% of MVC [34,35]. The energy supply is thus increasingly anaerobic, and the exertion had to be terminated [13,14,21,36].

P_max_ in our study was found at 37.21 ± 6.29% 1RM, which is close to the prescribed values from earlier work [13,14,21,25]. In terms of the maximum power delivered in the incremental test (5.38 ± 0.91 kg; 37.21% ± 6.29% 1RM), the percentages of LTP_1_ (35.32% ± 11.15%) and LTP_2_ (72.12% ± 14.13%) are comparable to results from incremental cycle ergometer tests [30,37], indicating some general patterns of metabolic regulation during incremental exercise. 

Both ventilatory thresholds VT_1_ and VT_2_ could be determined, although there were systemic ventilatory limits. Previous research revealed similar results for VT_2_ intensity with 24.37% ± 4.5% of 1RM [19] or 30.3% ± 7.9% of 1RM [14]. The rapid increase of breathing frequency (B_f_) and ventilation (VE) above LTP_2_ could be an attempt of respiratory compensation, failing due to a lack of venous return. It needs to be mentioned that terms and definitions of thresholds are unequivocal and sometimes mixed, which is misleading for the reader. Therefore, careful threshold definitions are warranted. For instance, two authors [13,20] used the term VT_1_ although all signs indicate a second threshold intensity. However, the overall pattern is comparable between their studies and our results.

Considering the pattern of the mean heart rate, an inverted deflection of the heart rate curve can be found [30,37], which was similar to other studies [14,21,25]. This pattern may be explained by a local muscular occlusion of blood supply altering venous return to the heart [32,36] as well as sympathetic drive [38]. If the load above LTP_2_ is continued, blood cannot be delivered and removed adequately from the muscle. Concomitant with this vascular occlusion, the heart attempts to compensate for the ongoing stress, but the increase in stroke volume indicated by the left ventricular ejection fraction (LVEF) is blunted because of the reduced venous return. This lack of preload of the heart is compensated by a marked increase in heart rate [29,36,39,40]. It is therefore suggested that even a load above LTP_2_ intensity, which in our case was just about 25% of the 1RM, already decreases blood supply to the working muscle limiting continuous exercise [14,36].

The subsequent results of the constant-load tests below and above LTP_1_ and LTP_2_ validated the concept of a lactate steady-state [4,8]. All tests below LTP_2_ demonstrated clear lactate steady-state but not above LTP_2_. We need to mention that the usual definition of a lactate steady-state of an increase of lactate concentration less than 1 mmol·L^−1^ during the last 20 min of a 30-min constant-load exercise [41,42] is difficult to apply in small muscle group exercise. Systemic lactate concentration is associated with working muscle mass involved. As shown in our study, small muscle exercise leads to low systemic lactate concentrations. The amount of produced lactate is distributed over the same blood volume as compared to exercise with larger muscle mass engaged [43]. We, therefore, adapted the definition of a lactate steady-state such as a maximal change of lactate concentration of no more than 0.2 mmol·L^−1^ during the last 20 min of a 30-min constant-load exercise was accepted. The mentioned definition of a lactate steady-state refers to higher maximal lactate concentrations reached during cycling loads (8–15 mmol·L^−1^). The idea was to create a more adequate ratio between lactate concentration fluctuation during the bicep curl constant-load exercise and maximal lactate concentration (2–3 mmol^−1^). During constant-load exercise below LTP_2_, a lactate steady state could be identified in all subjects by this definition; however, exercise just 5% of P_max_ above the LTP_2_ had to be terminated due to muscular failure within a short period of time, and no steady-state as defined was present. 

Heart rate during constant-load exercise below and above LTP_1_ was slightly above resting values, differed only marginally (*p* > 0.05), and both represented a steady-state of the heart rate. Besides the consequent application of a three-phase model for incremental and constant-load exercise, no data regarding small muscle group constant-load exercise below and above LTP_1_ intensity have been presented yet. In the constant-load exercise, just below the LTP_2_ heart rate increased by about 10 beats during the 30-min continuous load. A similar increase of about 6–8 heartbeats has been documented for steady-state cycle ergometer exercise below LTP_2_ [14,17]. This increase in HR even in small muscle group exercise is suggested to be caused by increasing body temperature, chronotropic effects such as an increased sympathetic drive [38], a baroreceptor mediated increase and/or a reduced parasympathetic tone [44,45,46]. An intensity above LTP_2_ could not be carried out for the full 30 min and was already terminated after about 16 min due to local muscle fatigue. However, the mentioned studies showed a different methodological approach, such as applied breaks between the exercise increments and added higher incremental load intensities (maximal intensity of 60% of 1RM). Furthermore, the repetitions per minute were in a range between 12 to 30 reps·min^−1^, and there was no measurement of LTP_1_ [13,14,15,16,17,18,19,20,21,22,23,24,25]. The higher incremental load intensities require resting intervals in between the sets, which indicates a typical resistance type exercise definition. To our knowledge, our study is the first to focus on the triphasic model of energy supply in small muscle exercise and the validation of LTP_1_ and LTP_2_ by means of constant-load exercise.

During the constant-load test below LTP_1_, VO_2_ and VCO_2_ remained at resting values for the entire 30 min of exercise. Such a low intensity of exercise below LTP_1_ did not show any signs of respiratory compensation, indicating that only the local muscular metabolism and the blood supply limit performance without reaching any systemic limits for ventilation. Above LTP_1_ VO_2_ and VCO_2_ settle after an initial rise and progress in a steady-state similar to exercise below LTP_2_. During exercise above LTP_2_, VCO_2_ concentration finally reached the VO_2_ (RER = 1), indicating an increasing local muscular anaerobic metabolism [47,48].

Measures of local metabolism and muscle recruitment need to be included in further studies as these measures provide additional, more accurate results and interpretations of the local muscle energetics and related hemodynamic processes [22]. As the percentages of 1RM were comparable with the literature, 1RM testing may be an easy tool not only to prescribe resistance exercise but also to prescribe constant-load endurance-type exercise such as investigated in our study. A more generalized view on training intensity prescription may be established in the future.

## 5. Conclusions

Our study is the first to show that the three-phase model of energy supply can even be applied and validated in a very small muscle mass incremental and constant-load bicep curl exercise. Both lactate turn points (LTP_1_/LTP_2_) and ventilatory thresholds (VT_1_/VT_2_) could be determined systemically during the incremental bicep curl exercise and constant-load exercise below and above these turn points, validating the concept of three-phase cardiorespiratory and metabolic response. The study showed that the three-phase model of energy supply [4] and the underlying lactate shuttle theory [2] are also indirectly applicable to small muscle mass exercise.

Thus, the lactate turn point concept [4], such as examined in our study, showed its applicability to and may serve as a basis for a more generalized model of training prescription, although additional studies including different muscle groups are still needed. Especially in the field of work physiology, this local muscular performance diagnostics could find a useful application. It helps to avoid overloading employees who are exposed to vigorous long term physical work and to set the balance between workload and resting intervals precisely. Another aspect is to prevent reduced performance levels at work and subsequent physical and/or mental health constraints.

## Figures and Tables

**Table 1:** Main characteristics of subjects.

Subjects	Sex	Age (years)	Height (cm)	Weight (kg)	BMI	1RM (kg)	VO_2max_ (Cycle) (mL·kg·min^−1^)
1	F	22.0	175.3	62.4	20.3	7.5	53.17
2	F	25.0	170.1	78.9	27.3	9.0	37.03
3	M	24.0	187.6	84.0	23.9	15.0	50.11
4	M	22.0	174.6	64.3	21.1	12.5	60.19
5	M	26.0	193.0	90.0	24.2	19.0	54.22
6	M	25.0	174.5	69.8	22.9	15.0	*
7	M	31.0	178.0	78.0	24.6	18.5	49.23
8	M	25.0	177.0	75.4	24.1	14.0	51.33
9	M	22.0	184.8	72.2	21.1	14.5	55.50
10	M	23.0	189.0	85.0	23.8	20.0	49.70
11	M	22.0	187.7	75.3	21.4	12.5	54.45
12	M	30.0	184.7	76.4	22.4	16.0	47.25

F: female; M: male; cm: centimeters; kg: kilograms; BMI: body mass index; VO_2max_: maximum oxygen uptake; mL·kg·min^−1^: milliliters per kilogram per minute; * no VO_2max_ measures were available for the cycle test.
